# 

**Published:** 1881-06

**Authors:** 


HERMAN MYNTER, M. D.

### CONTENTS:

PAGE.

Original Communications:

On the Treatment of Rotary-Lateral Curvature of the Spine. By Bernard Bartow, M. D., . . . . . . . . . 481

Gonorrhœal Rheumatism. By Herman Mynter, M. D., . . . . . . . 488

Clinical Reports:

Unusual Position of the Arteria Augnli Nasi. By Dr. Lucien Howe, . . . . . 495

Translations:

Microscopical Examination of Human Blood in a Case of Leukæmia Reported by MacGillavry, From the Dutch by P. W. Van Peyma, M. D., . . . . . 498

Selections:

Treatment of Anasarca, . . . . . 502

Food Adulteration, . . . . . . 503

Strychnia and Whiskey Hypodermically in Chloroform-Poisoning, . . . . . 504

Treatment of Cystitis, . . . . . . 505

Tight Rings, . . . . . . . . 505

Treatment and Pathology of Gout, . . . 506

Obstinate Vomiting in Pregnancy, . . . 507

Apocynum Cannabinum in Anasarca, . . 507

Alcohol in the Atmosphere, . . . . . 508

Color Blindness, . . . . . . . 508

Liebreich on Ozone, . . . . . . 509

Expiration of the Pylorus, . . . . . 513

Impotence from Salicylate of Soda, . . . 515

Society Reports:

New York State Pharmaceutical Association, 516

Editorial:

Our Public Institutions.—The State Insane Asylum, . . . . . . . . 519

Erie County Medical Society, . . . . 522

Reviews, . . . . . . . 522

